# Retinal Oxygen Extraction in Patients with Primary Open-Angle Glaucoma

**DOI:** 10.3390/ijms231710152

**Published:** 2022-09-05

**Authors:** Gerhard Garhöfer, Ahmed M. Bata, Alina Popa-Cherecheanu, Anton Hommer, Clemens Vass, Hemma Resch, Doreen Schmidl, René M. Werkmeister, Leopold Schmetterer

**Affiliations:** 1 Department of Clinical Pharmacology, Medical University of Vienna, 1090 Vienna, Austria; 2 Vienna Health Association, Landstrasse Hospital/Favoriten Hospital, 1030 Vienna, Austria; 3 Department of Ophthalmology, Carol Davila University of Medicine and Pharmacy District 5, 020021 Bucharest, Romania; 4 Department of Ophthalmology, Emergency University Hospital, 020021 Bucharest, Romania; 5 Department of Ophthalmology, Sanatorium Hera, 1090 Vienna, Austria; 6 Department of Ophthalmology, Medical University of Vienna, 1090 Vienna, Austria; 7 Center for Medical Physics and Biomedical Engineering, Medical University of Vienna, 1090 Vienna, Austria; 8 Singapore Eye Research Institute, Singapore 169856, Singapore; 9 Ophthalmology and Visual Sciences Academic Clinical Program, Duke-NUS Medical School, National University of Singapore, Singapore 169857, Singapore; 10 SERI-NTU Advanced Ocular Engineering (STANCE), Singapore 639798, Singapore; 11 School of Chemical and Biological Engineering, Nanyang Technological University Singapore, Singapore 639798, Singapore; 12 Institute of Molecular and Clinical Ophthalmology, 4031 Basel, Switzerland

**Keywords:** glaucoma, retinal oxygen extraction, retinal blood flow, Doppler optical coherence tomography

## Abstract

Objective: To compare total retinal oxygen extraction between patients with primary open-angle glaucoma (POAG) and healthy control subjects. Design: A prospective, single-center, cross-sectional, case–control study performed at the Medical University of Vienna. Subjects: Forty patients with POAG and 40 age- and sex-matched control subjects. Methods: Total retinal blood flow was measured using Doppler optical coherence tomography (OCT). Retinal arterial and venous oxygen saturation was measured using reflectance spectroscopy. From these parameters, oxygen content in the retinal arterial and venous circulation as well as total retinal oxygen extraction were calculated. Results: Total retinal blood flow was lower in POAG (25.2 ± 6.7 µL/min) as compared to healthy control subjects (35.6 ± 8.3 µL/min, *p* < 0.001). Retinal arterial oxygen content was not different between the two groups (0.18 ± 0.01 mL(O2)/mL in both groups, *p* < 0.761), but retinal venous oxygen content was higher in POAG (0.15 ± 0.01 mL(O2)/mL) than in healthy controls (0.14 ± 0.01 mL(O2)/mL *p* < 0.001). Accordingly, retinal oxygen extraction was reduced in POAG (0.8 ± 0.3 µL(O2)/min as compared to healthy controls: 1.4 ± 0.4 µL(O2)/min, *p* < 0.001). There was a significant association between total retinal blood flow and total retinal oxygen extraction with measures of structural and functional damage (*p* < 0.001 each). Conclusions: This study indicates that POAG is associated with a reduction in total retinal oxygen extraction linked to structural and functional damage of the disease. Since the technology is non-invasive, it allows for longitudinal studies investigating to which degree low retinal oxygen extraction is linked to the progression of the disease.

## 1. Introduction

Glaucoma is a chronic disease characterized by progressive optic nerve head (ONH) damage and loss of retinal ganglion cells (RGCs), leading to visual field defects. The main risk factor for glaucoma is elevated intraocular pressure (IOP). Reducing IOP slows down the progression of the disease, as shown in several large multicenter trials [1,2,3]. Some patients, however, still progress despite adequately controlled IOP. For a long period, it has been speculated that vascular factors play a role in the processes that lead to glaucomatous damage [4,5]. Measuring blood flow in the human eye is, however, not an easy task, and with most techniques, both validity and reproducibility are issues [6,7,8,9].

In recent years, efforts have been made to quantify blood flow using Doppler optical coherence tomography (OCT), and several groups have presented techniques to quantify total retinal blood flow [10,11,12,13,14]. We developed a dual-beam bidirectional Doppler OCT system to quantify blood flow [15] and have validated this technology by measurements at vessel bifurcations [16], comparison with laser Doppler velocimetry in humans [17], and by comparison with fluorescent microspheres in non-human primates [18]. In addition, we have coupled the system to a commercially available fundus camera-based system for the measurement of oxygen saturation in retinal vessels [19]. Based on mathematical modeling, this allows for the measurement of total retinal oxygen extraction [20].

In the present study, we hypothesized that total retinal blood flow and total retinal oxygen extraction are reduced in patients with primary open-angle glaucoma (POAG). To test this hypothesis, we performed a cross-sectional study in 40 patients with POAG and in 40 age- and sex-matched healthy control subjects. To better understand the relation to the disease process, we correlated hemodynamic changes to structural and functional measures of glaucomatous damage.

## 2. Results

The characteristics of the recruited subjects are presented in Table 1. Subjects were matched for age and sex and no differences in blood pressure and pulse rate were observed. As expected, RNFLT was significantly lower in glaucoma patients as compared to healthy controls (*p* < 0.001).

**Figure 1:**
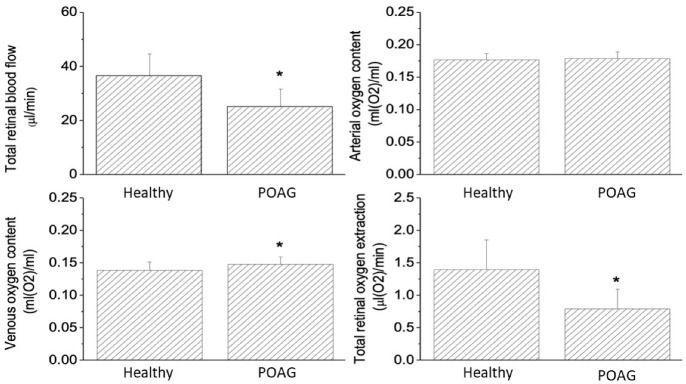
Retinal hemodynamic parameters in healthy control subjects and patients with POAG. Data are presented as means ± SD. * significant difference between the two groups.

Figure 1 shows the comparison of retinal hemodynamic parameters between patients with POAG and healthy controls. Total retinal blood flow was reduced in PAOG patients as compared to healthy controls (POAG: 25.2 ± 6.7 μL/min, healthy controls: 35.6 ± 8.3 μL/min *p* < 0.001). Whereas retinal arterial oxygen content was not different between the two groups (POAG: 0.18 ± 0.01 mL(O2)/mL, healthy controls: 0.18 ± 0.01 mL(O2)/mL, *p* = 0.761), retinal venous oxygen content was higher in PAOG as compared to healthy controls (POAG: 0.15 ± 0.01 mL(O2)/mL, healthy controls: 0.14 ± 0.01 mL(O2)/mL, *p* < 0.001). Since the arterio-venous oxygen content difference (POAG: 0.03 ± 0.01 mL(O2)/mL, healthy controls (0.04 ± 0.01 mL(O2)/mL, *p* < 0.001) as well as the total retinal blood flow were reduced in POAG, we also observed a lower total retinal oxygen extraction in POAG (POAG: 0.8 ± 0.3 μL(O2)/min, healthy controls: 1.4 ± 0.4 μL(O2)/min, *p* < 0.001).

**Figure 2:**
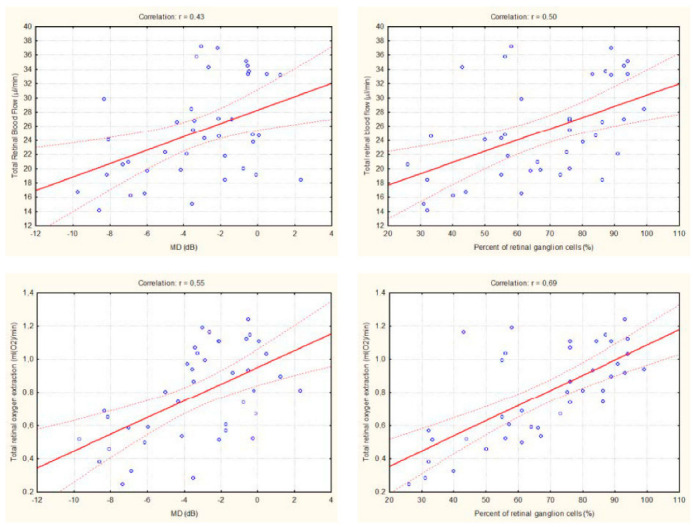
Linear correlation between retinal hemodynamic parameters and measures of structural damage in POAG patients.

Figure 2 shows the correlation between structural and functional glaucoma measures and hemodynamic parameters. Significant associations were observed between total retinal blood flow with both MD and percentage of RGCs (*p* < 0.001 each). The correlation between total retinal oxygen extraction and MD as well as percentage of RGCs was even higher (*p* < 0.001 each). The highest correlation coefficient was observed between retinal oxygen extraction and percentage of RGCs (r = 0.69). In glaucoma eyes RNFLT was significantly associated with total retinal blood flow (r = 0.41, *p* < 0.01) and total retinal oxygen extraction (r = 0.51, *p* < 0.01). In glaucomatous eyes no significant correlation was observed between total retinal blood flow and IOP (r = 0.07, *p* = 0.67) or total retinal oxygen extraction and IOP (r = 0.14, *p* = 0.39).

## 3. Discussion

The current study shows that retinal oxygen extraction is reduced in patients with glaucoma and is strongly correlated to the associated structural and functional damage. This is in keeping with a previous smaller-scale study in glaucoma patients [21]. Whereas several previous studies have shown that there is an association between ONH and/or retinal blood flow with glaucoma damage [22,23,24,25,26,27], our study links RGC damage to oxygen metabolism.

Whether the changes in blood flow and oxygen metabolism in glaucoma are the cause or the consequence of glaucomatous damage is a long-standing discussion. OCT angiography studies have consistently demonstrated a reduction in capillary density in the macular region as well as in the peripapillary region in patients with early and manifest glaucoma [28,29]. Recently, it has been reported that there is a strong association between local perfusion defects and local visual field changes in glaucoma [30,31,32,33,34], indicating that at least part of the vascular changes is the result of reduced metabolic demand secondary to retinal ganglion cell loss.

There are, however, several lines of evidence that the reduction in retinal blood flow and oxygen metabolism is causative and contributes to disease progression. Four studies using color Doppler imaging to measure retrobulbar arterial blood velocities reported that reduced values were associated with visual field progression of the disease [35,36,37,38]. More recently, studies using either OCT angiography [39,40] or laser speckle flowgraphy indicated similar results [41]. In addition, two studies reported an increase in retinal blood flow in very early glaucoma cases due to an unknown reason, potentially indicating a counter-regulatory mechanism to promote retinal ganglion cell survival [42,43]. Whether this would also be associated with increased oxygen extraction remains to be investigated. Interestingly, we found in patients with early diabetes with no or mild diabetic retinopathy that total retinal blood flow is increased, but retinal oxygen extraction is decreased [44].

The results obtained from total retinal blood flow in the present paper are in good agreement with previous papers quantifying retinal perfusion using either laser Doppler velocimetry or Doppler OCT for the quantification of blood velocity [10,11,12,45,46,47,48,49]. Currently, however, none of the techniques for measuring total retinal blood flow has been commercialized, and the technique is not clinically available. Retinal oximetry as used in the present study to measure oxygen saturation in retinal vessels is commercially available, and our results are in agreement with findings from other groups [50,51,52,53], but data are difficult to interpret if blood flow is not measured concomitantly [54,55].

The strengths and limitations of the present study require attention. The strength of the current study is the use of state-of-the-art techniques for the non-invasive, non-contractile measurement of oxygen extraction. Based on reflectometric measurements of the oxygen saturation in retinal vessels together with the determination of total retinal blood flow using Doppler optical coherence tomography, we can draw conclusions on the oxygen metabolism of the retina without invasive measurements. We have successfully used this approach in previous studies to investigate oxygen metabolisms in healthy subjects [55,56] as well as in pathological conditions [44,57]. As all techniques used in the current study are non-invasive and thus well tolerated, this approach is also suitable for larger studies.

In this pilot study, however, the sample size is relatively small, and conformation in a larger study population requires attention. As mentioned above, the study is cross-sectional and it is unknown to which degree low retinal oxygen extraction is related to glaucoma progression. Thus, based on the data available, the question of whether the observed change in oxygen metabolism is a cause or consequence of the disease cannot finally be answered. A longitudinal study will be necessary to further investigate this issue. The technology used in the present study is not capable of measuring tissue oxygen extraction in vivo but is limited to larger retinal vessels. As such, sectoral analysis of the relationship between visual field defects and reduced oxygen metabolism is not possible.

In conclusion, the present study indicates reduced retinal oxygen extraction in patients with glaucoma associated with both functional and structural damage. Longitudinal studies are required to understand the relation to glaucoma progression.

## 4. Methods and Materials

### 4.1. Subjects

The present study was approved by the Ethics Committee of the Medical University of Vienna. It was performed according to the guidelines specified in the Declaration of Helsinki and the Good Clinical Practice (GCP) guidelines. A total of 40 patients with POAG and 40 healthy control subjects were included in this cross-sectional study. All participating subjects passed a screening examination that included physical examination, measurement of systemic blood pressure as well as an ophthalmic examination. Diagnosis of manifest POAG was defined as pathological optic disc appearance and glaucoma hemifield test outside normal limits.

Exclusion criteria were mean deviation in the visual field test >10 dB, pseudoexfoliation glaucoma, pigmentary glaucoma, secondary glaucoma, evidence of angle closure, intraocular surgery within the last six months, diabetes mellitus, and untreated hypertension with systolic blood pressure > 160 mmHg, diastolic blood pressure > 95 mmHg, abuse of alcoholic beverages, presence or history of a severe medical condition as judged by the investigator, and participation in a clinical trial in the three weeks preceding the study. Control subjects were age- and sex-matched with normal ophthalmic findings and IOP < 21 mmHg on at least three measurements, no evidence of increased IOP in the medical history, and no signs of glaucomatous damage in the optic disc or as revealed via OCT. Intraocular pressure was measured using Goldmann applanation tonometry. For measurements, the pupil was dilated using one drop of 0.5% tropicamide (Mydriaticum “Agepha”, Agepha, Vienna, Austria). Measurements were done after a resting period of 20 min to achieve stable hemodynamic conditions.

### 4.2. Methods

#### 4.2.1. Measurement of Total Retinal Blood Flow

The technique for measuring total retinal blood flow has been described previously [12]. Briefly, we use a custom-built dual-beam bidirectional Fourier-domain Doppler OCT system [15]. The use of two light beams illuminating the retina under two different angles offers the advantage that the angle ambiguity in the Doppler equation can be overcome and allows for measurement of absolute blood velocity, vessel diameter [58], and retinal blood flow [16,17]. In the present study, we used a recently published algorithm for retinal blood velocities [49]. In order to obtain total retinal blood flow (Q), measurements of blood flow were taken from all arteries and all veins entering the optic nerve head (retinal arteries: blood velocities v_i_, vessel diameters d*_i_*, blood flow: Q_A,tot_; retinal veins: blood velocities v_j_, vessel diameters d*_j_*_,_ blood flow: Q_V,tot_ ).

$$Q_{A,\;\mathrm{tot}}=\sum_{i=1}^{\#A}Q_{A,i},$$

$$Q_{V,\;\mathrm{tot}}=\sum_{j=1}^{\#V}Q_{V,j}.$$

Because the retina is an end organ, total retinal blood flow needs to equal when either obtained from arteries or from veins. Total retinal blood flow is therefore calculated as the mean between values as obtained from retinal arteries and retinal veins, respectively.

$$Q=\frac{Q_{A,\mathrm{tot}}+Q_{V,\mathrm{tot}}}{2}.$$

#### 4.2.2. Measurement of Retinal Oxygen Saturation

In order to allow for the calculation of total retinal oxygen extraction, the OCT system is coupled to the commercially available Dynamic Vessel Analyzer (DVA, Imedos, Jena, Germany). This system includes an oxygen module for fundus camera-based oxygen saturation measurements in retinal arteries and veins [19]. Fundus photographs are concomitantly taken at two different wavelengths (610 and 545 nm). Since oxygenated and deoxygenated hemoglobin have different light absorption characteristics, this allows for calculation of retinal oxygen saturation (*SaO*_2_*)*. At 545 nm, which is close to the isobestic point (548 nm), the absorption for oxygenated and deoxygenated hemoglobin is almost equal. At a wavelength of 610 nm, oxygenated hemoglobin shows very little absorption and is nearly transparent. In the present study, oxygen saturation was measured in all retinal arteries (*SaO*_2,A_)*_i_*, and all retinal veins *(SaO*_2*,V*_*)_j_* at the same locations where blood flow measurement was performed. A sample measurement is shown in Supplementary Figure S1.

#### 4.2.3. Calculation of Total Retinal Oxygen Extraction

We have previously established a mathematical model to calculate total retinal oxygen extraction based on these measurements [20]. The idea of this model is to calculate oxygen content and blood flow in the central retinal artery and the central retinal vein at the level where they enter the ONH. To do so, one has to correct for the oxygen loss through the vascular wall between the entrance of the vessel at the optic disc and the measurement sites of the branch arteries and branch veins. In addition, the model accounts for differences in oxygen saturation between different branch veins that merge into the central retinal vein. Finally, our original model took into account that oxygen is partially physically dissolved in plasma. To account for this, we measured oxygen tension (pO_2_) from an arterialized blood sample taken from the earlobe and determined hematocrit from a venous blood sample. In the present study, we did not collect blood samples and as such have estimated this portion based on a pO_2_ level of 100 mg and a hematocrit of 46% in men and 42% in women. The error that arises from this limitation is <1%. We then calculated oxygen content at the level of the central retinal artery (cO_2,CRA_) and the central retinal vein (cO_2,CRV_) and total retinal oxygen extraction as:

(1) $$extO_{2}=(cO_{2,\mathrm{CRA}}-cO_{2,\mathrm{CRV}})\cdot Q.$$

#### 4.2.4. Measurement of Retinal Nerve Fiber Layer Thickness

Retinal nerve fiber layer thickness (RNFLT) was measured using a commercially available spectral domain OCT system (SD-OCT (Heidelberg Spectralis OCT, SPECTRALIS software version 5.3.3.0, EYE EXPLORER Software 1.6.4.0; Heidelberg Engineering, Heidelberg, Germany).

#### 4.2.5. Estimation of Retinal Ganglion Cell Number

The model proposed by Harwerth and co-workers was used to estimate the total number of retinal ganglion cells [59]. This association is based on the measurement of structural and functional data in non-human primates and comparison with histology-derived retinal ganglion counts. The model accounts for age-related loss of axonal density and for glaucoma stage-dependent alterations in the relationship between the neuronal and non-neuronal components of retinal tissues as measured with OCT. We have followed this approach in a previous paper investigating the age-related decline of retinal oxygen extraction in healthy subjects [60]. In the present paper, the percentage of RGCs in glaucoma patients was calculated as the ratio of the number of RGCs in glaucoma patients/mean of RGCs in the healthy control subjects.

#### 4.2.6. Measurement of Blood Pressure and Pulse Rate

Systolic, diastolic, and mean arterial blood pressures (SBP, DBP, MAP) were measured on the upper arm by an automated oscillometric device (HP-CMS patient monitor; Hewlett Packard, Palo Alto, CA, USA). Pulse rate was automatically recorded by the same device from a finger pulse oximetry device.

#### 4.2.7. Statistical Analysis

Data are presented as means ± standard deviation (SD). Differences between patients with glaucoma and healthy control subjects were studied using an unpaired t-test. Linear correlation analysis was used to study the association between hemodynamic measures on the one hand and functional and structural data on the other hand. A value of *p* < 0.05 was considered as the level of significance.

## Figures and Tables

**Table 1:** Characteristics of glaucoma patients and matched healthy.

	POAG	Healthy Controls	*p*-Value *
Age (years)	58 ± 8	57 ± 7	0.687
Sex (male/female)	14/26	14/26	-
Intraocular pressure (mmHg)	16 ± 3	15 ± 3	0.826
Systolic blood pressure (mmHg)	132 ± 10	129 ± 9	0.211
Diastolic blood pressure (mmHg)	72 ± 8	70 ± 7	0.336
Mean arterial pressure (mmHg)	92 ± 8	90 ± 7	0.259
Pulse rate (beats/min)	67 ± 10	65 ± 9	0.417
Intraocular Pressure (mmHg)	17 ± 3	16 ± 3	0.677
Retinal nerve fiber layer thickness (µm)	73 ± 15	99 ± 8	**<0.001**
Mean deviation (MD)	−6.3 ± 3.3	-	-

Data except for sex are presented as means ± SD, * significant difference between the two groups (unpaired *t*-test).

## Data Availability

The data presented in this study are available on request from the corresponding author.

## Supplementary Materials

The following supporting information can be downloaded at: https://www.mdpi.com/article/10.3390/ijms231710152/s1.

## References

[1] Heijl A., Leske M.C., Bengtsson B., Hyman L., Bengtsson B., Hussein M., Early Manifest Glaucoma Trial G. (2002). Reduction of intraocular pressure and glaucoma progression: Results from the Early Manifest Glaucoma Trial. Arch. Ophthalmol..

[2] Schmidl D., Schmetterer L., Garhöfer G., Popa-Cherecheanu A. (2015). Pharmacotherapy of Glaucoma. J. Ocul. Pharmacol. Ther..

[3] Garway-Heath D.F., Crabb D.P., Bunce C., Lascaratos G., Amalfitano F., Anand N., Azuara-Blanco A., Bourne R.R., Broadway D.C., A Cunliffe I. (2015). Latanoprost for open-angle glaucoma (UKGTS): A randomised, multicentre, placebo-controlled trial. Lancet.

[4] Flammer J., Orgül S., Costa V.P., Orzalesi N., Krieglstein G.K., Serra L.M., Renard J.-P., Stefánsson E. (2002). The impact of ocular blood flow in glaucoma. Prog. Retin. Eye Res..

[5] Cherecheanu A.P., Garhofer G., Schmidl D., Werkmeister R., Schmetterer L. (2013). Ocular perfusion pressure and ocular blood flow in glaucoma. Curr. Opin. Pharmacol..

[6] Schmetterer L., Garhofer G. (2007). How can blood flow be measured?. Surv. Ophthalmol..

[7] Harris A., Kagemann L., Ehrlich R., Rospigliosi C., Moore D., Siesky B. (2008). Measuring and interpreting ocular blood flow and metabolism in glaucoma. Can. J. Ophthalmol..

[8] Sadda S.R., Maram J., Srinivas S. (2017). Evaluating ocular blood flow. Indian J. Ophthalmol..

[9] Wei X., Balne P.K., Meissner K.E., Barathi V.A., Schmetterer L., Agrawal R. (2018). Assessment of flow dynamics in retinal and choroidal microcirculation. Surv. Ophthalmol..

[10] Wang Y., Lu A., Gil-Flamer J., Tan O., A Izatt J., Huang D. (2009). Measurement of total blood flow in the normal human retina using Doppler Fourier-domain optical coherence tomography. Br. J. Ophthalmol..

[11] Baumann B., Potsaid B., Kraus M.F., Liu J.J., Huang D., Hornegger J., Cable A.E., Duker J.S., Fujimoto J.G. (2011). Total retinal blood flow measurement with ultrahigh speed swept source/Fourier domain OCT. Biomed. Opt. Express.

[12] Doblhoff-Dier V., Schmetterer L., Vilser W., Garhöfer G., Gröschl M., Leitgeb R.A., Werkmeister R.M. (2014). Measurement of the total retinal blood flow using dual beam Fourier-domain Doppler optical coherence tomography with orthogonal detection planes. Biomed. Opt. Express.

[13] Haindl R., Trasischker W., Wartak A., Baumann B., Pircher M., Hitzenberger C.K. (2016). Total retinal blood flow measurement by three beam Doppler optical coherence tomography. Biomed. Opt. Express.

[14] Tani T., Song Y.-S., Yoshioka T., Omae T., Ishibazawa A., Akiba M., Yoshida A. (2017). Repeatability and Reproducibility of Retinal Blood Flow Measurement Using a Doppler Optical Coherence Tomography Flowmeter in Healthy Subjects. Investig. Opthalmol. Vis. Sci..

[15] Werkmeister R.M., Dragostinoff N., Pircher M., Götzinger E., Hitzenberger C.K., Leitgeb R.A., Schmetterer L. (2008). Bidirectional Doppler Fourier-domain optical coherence tomography for measurement of absolute flow velocities in human retinal vessels. Opt. Lett..

[16] Werkmeister R.M., Dragostinoff N., Palkovits S., Told R., Boltz A., Leitgeb R.A., Gröschl M., Garhöfer G., Schmetterer L. (2012). Measurement of Absolute Blood Flow Velocity and Blood Flow in the Human Retina by Dual-Beam Bidirectional Doppler Fourier-Domain Optical Coherence Tomography. Investig. Opthalmol. Vis. Sci..

[17] Werkmeister R.M., Palkovits S., Told R., Gröschl M., Leitgeb R.A., Garhöfer G., Schmetterer L. (2012). Response of Retinal Blood Flow to Systemic Hyperoxia as Measured with Dual-Beam Bidirectional Doppler Fourier-Domain Optical Coherence Tomography. PLoS ONE.

[18] Told R., Wang L., Cull G., Thompson S.J., Burgoyne C.F., Aschinger G.C., Schmetterer L., Werkmeister R.M. (2016). Total Retinal Blood Flow in a Nonhuman Primate Optic Nerve Transection Model Using Dual-Beam Bidirectional Doppler FD-OCT and Microsphere Method. Investig. Opthalmol. Vis. Sci..

[19] Hammer M., Vilser W., Riemer T., Schweitzer D. (2008). Retinal vessel oximetry-calibration, compensation for vessel diameter and fundus pigmentation, and reproducibility. J. Biomed. Opt..

[20] Werkmeister R.M., Schmidl D., Aschinger G., Doblhoff-Dier V., Palkovits S., Wirth M., Garhöfer G., Linsenmeier R.A., Leitgeb R., Schmetterer L. (2015). Retinal oxygen extraction in humans. Sci. Rep..

[21] A Aref A., Maleki S., Tan O., Huang D., Varma R., Shahidi M. (2019). Relating glaucomatous visual field loss to retinal oxygen delivery and metabolism. Acta Ophthalmol..

[22] Yoshioka T., Song Y., Kawai M., Tani T., Takahashi K., Ishiko S., Lavinsky F., Wollstein G., Ishikawa H., Schuman J.S. (2020). Retinal blood flow reduction in normal-tension glaucoma with single-hemifield damage by Doppler optical coherence tomography. Br. J. Ophthalmol..

[23] Sehi M., Goharian I., Konduru R., Tan O., Srinivas S., Sadda S.R., Francis B.A., Huang D., Greenfield D.S. (2013). Retinal Blood Flow in Glaucomatous Eyes with Single-Hemifield Damage. Ophthalmology.

[24] Hwang J., Konduru R., Zhang X., Tan O., A Francis B., Varma R., Sehi M., Greenfield D.S., Sadda S.R., Huang D. (2012). Relationship among Visual Field, Blood Flow, and Neural Structure Measurements in Glaucoma. Investig. Opthalmol. Vis. Sci..

[25] Deokule S., Vizzeri G., Boehm A., Bowd C., Weinreb R.N. (2010). Association of Visual Field Severity and Parapapillary Retinal Blood Flow in Open-Angle Glaucoma. J. Glaucoma.

[26] Yamada Y., Higashide T., Udagawa S., Takeshima S., Sakaguchi K., Nitta K., Sugiyama K. (2019). The Relationship Between Interocular Asymmetry of Visual Field Defects and Optic Nerve Head Blood Flow in Patients with Glaucoma. J. Glaucoma.

[27] Resch H., Schmidl D., Hommer A., Rensch F., Jonas J.B., Fuchsjäger-Mayrl G., Garhöfer G., Vass C., Schmetterer L. (2011). Correlation of optic disc morphology and ocular perfusion parameters in patients with primary open angle glaucoma. Acta Ophthalmol..

[28] Fan X., Ying Y., Zhai R., Sheng Q., Sun Y., Xu H., Kong X. (2022). The characteristics of fundus microvascular alterations in the course of glaucoma: A narrative review. Ann. Transl. Med..

[29] Lee A., Sung K.R., Shin J.W. (2022). Progression detection capabilities of circumpapillary and macular vessel density in advanced glaucomatous eyes. Sci. Rep..

[30] Calzetti G., Mursch-Edlmayr A.S., Bata A.M., Ungaro N., Mora P., Chua J., Schmidl D., Bolz M., Garhöfer G., Gandolfi S. (2021). Measuring optic nerve head perfusion to monitor glaucoma: A study on structure–function relationships using laser speckle flowgraphy. Acta Ophthalmol..

[31] Kallab M., Hommer N., Schlatter A., Chua J., Tan B., Schmidl D., Hirn C., Findl O., Schmetterer L., Garhöfer G. (2022). Combining vascular and nerve fiber layer thickness measurements to model glaucomatous focal visual field loss. Ann. New York Acad. Sci..

[32] Wong D., Chua J., Lin E., Tan B., Yao X., Chong R., Sng C., Lau A., Husain R., Aung T. (2020). Focal Structure–Function Relationships in Primary Open-Angle Glaucoma Using OCT and OCT-A Measurements. Investig. Opthalmol. Vis. Sci..

[33] Wong D., Chua J., Tan B., Yao X., Chong R., Sng C.C.A., Husain R., Aung T., Garway-Heath D., Schmetterer L. (2021). Combining OCT and OCTA for Focal Structure–Function Modeling in Early Primary Open-Angle Glaucoma. Investig. Opthalmol. Vis. Sci..

[34] Yaoeda K., Shirakashi M., Fukushima A., Funaki S., Funaki H., Abe H., Tanabe N. (2003). Relationship between optic nerve head microcirculation and visual field loss in glaucoma. Acta Ophthalmol. Scand..

[35] Galassi F., Sodi A., Ucci F., Renieri G., Pieri B., Baccini M. (2003). Ocular hemodynamics and glaucoma prognosis: A color Doppler imaging study. Arch. Ophthalmol..

[36] Satilmis M., Orgül S., Doubler B., Flammer J. (2003). Rate of progression of glaucoma correlates with retrobulbar circulation and intraocular pressure. Am. J. Ophthalmol..

[37] Siesky B., Harris A., Carr J., Vercellin A.V., Hussain R.M., Hembree P.P., Wentz S., Isaacs M., Eckert G., Moore N.A. (2016). Reductions in Retrobulbar and Retinal Capillary Blood Flow Strongly Correlate with Changes in Optic Nerve Head and Retinal Morphology over 4 Years in Open-angle Glaucoma Patients of African Descent Compared with Patients of European Descent. J. Glaucoma.

[38] Zeitz O., Galambos P., Wagenfeld L., Wiermann A., Wlodarsch P., Praga R., Matthiessen E.T., Richard G., Klemm M. (2006). Glaucoma progression is associated with decreased blood flow velocities in the short posterior ciliary artery. Br. J. Ophthalmol..

[39] Jeon S.J., Shin D.-Y., Park H.-Y.L., Park C.K. (2019). Association of Retinal Blood Flow with Progression of Visual Field in Glaucoma. Sci. Rep..

[40] Wang Y.M., Shen R., Lin T.P., Chan P.P., Wong M.O., Chan N.C., Tang F., Lam A.K., Leung D.Y., Tham C.C. (2022). Optical coherence tomography angiography metrics predict normal tension glaucoma progression. Acta Ophthalmol..

[41] Kiyota N., Shiga Y., Omodaka K., Pak K., Nakazawa T. (2020). Time-Course Changes in Optic Nerve Head Blood Flow and Retinal Nerve Fiber Layer Thickness in Eyes with Open-angle Glaucoma. Ophthalmology.

[42] Berisha F., Feke G.T., Hirose T., McMeel J.W., Pasquale L.R. (2008). Retinal Blood Flow and Nerve Fiber Layer Measurements in Early-Stage Open-Angle Glaucoma. Am. J. Ophthalmol..

[43] Gardiner S.K., Cull G., Fortune B., Wang L. (2019). Increased Optic Nerve Head Capillary Blood Flow in Early Primary Open-Angle Glaucoma. Investig. Opthalmol. Vis. Sci..

[44] Fondi K., Wozniak P.A., Howorka K., Bata A.M., Aschinger G.C., Popa-Cherecheanu A., Witkowska K.J., Hommer A., Schmidl D., Werkmeister R.M. (2017). Retinal oxygen extraction in individuals with type 1 diabetes with no or mild diabetic retinopathy. Diabetologia.

[45] E Riva C., E Grunwald J., Sinclair S.H., Petrig B.L. (1985). Blood velocity and volumetric flow rate in human retinal vessels. Investig. Ophthalmol. Vis. Sci..

[46] Polska E., Kircher K., Ehrlich P., Vecsei P.V., Schmetterer L. (2001). RI in central retinal artery as assessed by CDI does not correspond to retinal vascular resistance. Am. J. Physiol. Circ. Physiol..

[47] Garhofer G., Werkmeister R., Dragostinoff N., Schmetterer L. (2012). Retinal Blood Flow in Healthy Young Subjects. Investig. Opthalmol. Vis. Sci..

[48] Rose K., Flanagan J.G., Patel S.R., Cheng R., Hudson C. (2014). Retinal Blood Flow and Vascular Reactivity in Chronic Smokers. Investig. Opthalmol. Vis. Sci..

[49] Szegedi S., Hommer N., Kallab M., Puchner S., Schmidl D., Werkmeister R.M., Garhöfer G., Schmetterer L. (2020). Repeatability and Reproducibility of Total Retinal Blood Flow Measurements Using Bi-Directional Doppler OCT. Transl. Vis. Sci. Technol..

[50] Olafsdottir O.B., Hardarson S.H., Gottfredsdottir M.S., Harris A., Stefánsson E. (2011). Retinal Oximetry in Primary Open-Angle Glaucoma. Investig. Opthalmol. Vis. Sci..

[51] Shahidi A.M., Hudson C., Tayyari F., Flanagan J.G. (2016). Retinal Oxygen Saturation in Patients with Primary Open-angle Glaucoma Using a Non-flash Hypespectral Camera. Curr. Eye Res..

[52] Vandewalle E., Pinto L.A., Olafsdottir O.B., De Clerck E., Stalmans P., Van Calster J., Zeyen T., Stefánsson E., Stalmans I. (2013). Oximetry in glaucoma: Correlation of metabolic change with structural and functional damage. Acta Ophthalmol..

[53] Yap Z.L., Ong C., Lee Y.F., Tsai A., Cheng C., Nongpiur M.E., Perera S. (2017). Retinal Oximetry in Subjects with Glaucomatous Hemifield Asymmetry. J. Glaucoma.

[54] Palkovits S., Lasta M., Told R., Schmidl D., Boltz A., Napora K.J., Werkmeister R.M., Popa-Cherecheanu A., Garhöfer G., Schmetterer L. (2014). Retinal Oxygen Metabolism During Normoxia and Hyperoxia in Healthy Subjects. Investig. Opthalmol. Vis. Sci..

[55] Palkovits S., Told R., Schmidl D., Boltz A., Napora K.J., Lasta M., Kaya S., Werkmeister R.M., Popa-Cherecheanu A., Garhöfer G. (2014). Regulation of retinal oxygen metabolism in humans during graded hypoxia. Am. J. Physiol. Circ. Physiol..

[56] Palkovits S., Lasta M., Told R., Schmidl D., Werkmeister R., Cherecheanu A.P., Garhöfer G., Schmetterer L. (2015). Relation of retinal blood flow and retinal oxygen extraction during stimulation with diffuse luminance flicker. Sci. Rep..

[57] Kallab M., Hommer N., Schlatter A., Bsteh G., Altmann P., Popa-Cherecheanu A., Pfister M., Werkmeister R.M., Schmidl D., Schmetterer L. (2021). Retinal Oxygen Metabolism and Haemodynamics in Patients with Multiple Sclerosis and History of Optic Neuritis. Front. Neurosci..

[58] Fondi K., Aschinger G.C., Bata A.M., Wozniak P.A., Liao L., Seidel G., Doblhoff-Dier V., Schmidl D., Garhöfer G., Werkmeister R.M. (2016). Measurement of Retinal Vascular Caliber From Optical Coherence Tomography Phase Images. Investig. Opthalmol. Vis. Sci..

[59] Harwerth R., Wheat J., Fredette M., Anderson D. (2010). Linking structure and function in glaucoma. Prog. Retin. Eye Res..

[60] Bata A.M., Fondi K., Szegedi S., Aschinger G.C., Hommer A., Schmidl D., Chua J., Werkmeister R.M., Garhöfer G., Schmetterer L. (2019). Age-Related Decline of Retinal Oxygen Extraction in Healthy Subjects. Investig. Opthalmol. Vis. Sci..

