# Supplementary material for: Retinal Oxygen Extraction in Patients with Primary Open-Angle Glaucoma
Source: Int J Mol Sci. 2022 Sep 5;23(17):10152. doi: 10.3390/ijms231710152 (PMC9456494; doi:10.3390/ijms231710152)
Supplement: Supplementary file 1 [file ijms-23-10152-s001.zip › ijms-1837071-supplementary.pdf]

Supplemental Figure S1

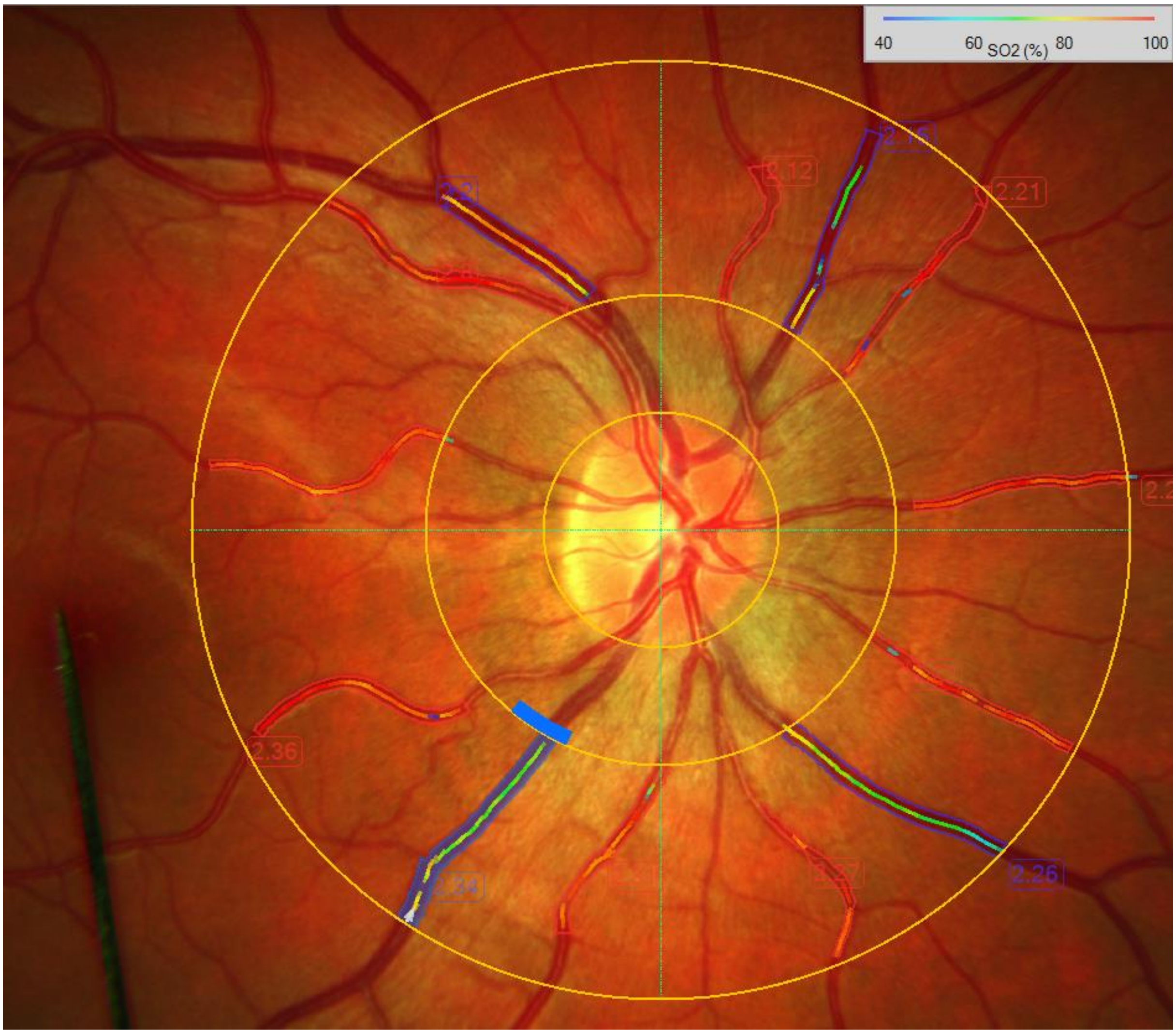

Non-invasive measurement of retinal oxygen saturation using retinal oximetry. The color scale on the right top of the image indicates oxygen saturation in percent. The retinal arterioles appear generally red indicating oxygen saturation in the 90–100% range, whereas the retinal veins are green/yellow/blue indicating lower oxygen saturation compared to the retinal arteries.
